# A Systematic Review and Meta-Analysis Comparing the Diagnostic Accuracy Tests of COVID-19

**DOI:** 10.3390/diagnostics13091549

**Published:** 2023-04-26

**Authors:** Juan Jeferson Vilca-Alosilla, Mayron Antonio Candia-Puma, Katiusca Coronel-Monje, Luis Daniel Goyzueta-Mamani, Alexsandro Sobreira Galdino, Ricardo Andrez Machado-de-Ávila, Rodolfo Cordeiro Giunchetti, Eduardo Antonio Ferraz Coelho, Miguel Angel Chávez-Fumagalli

**Affiliations:** 1 Computational Biology and Chemistry Research Group, Vicerrectorado de Investigación, Universidad Católica de Santa María, Arequipa 04000, Peru; 2 Facultad de Ciencias Farmacéuticas, Bioquímicas y Biotecnológicas, Universidad Católica de Santa María, Arequipa 04000, Peru; 3 Sustainable Innovative Biomaterials Department, Le Qara Research Center, Arequipa 04000, Peru; 4 Laboratório de Biotecnologia de Microrganismos, Universidade Federal São João Del-Rei, Divinópolis 35501-296, MG, Brazil; 5 Programa de Pós-Graduação em Ciências da Saúde, Universidade do Extremo Sul Catarinense, Criciúma 88806-000, SC, Brazil; 6 Laboratório de Biologia das Interações Celulares, Instituto de Ciências Biológicas, Universidade Federal de Minas Gerais, Belo Horizonte 31270-901, MG, Brazil; 7 Instituto Nacional de Ciência e Tecnologia em Doenças Tropicais, INCT-DT, Salvador 40015-970, BA, Brazil; 8 Programa de Pós-Graduação em Ciências da Saúde: Infectologia e Medicina Tropical, Faculdade de Medicina, Universidade Federal de Minas Gerais, Belo Horizonte 31270-901, MG, Brazil; 9 Departamento de Patologia Clínica, COLTEC, Universidade Federal de Minas Gerais, Belo Horizonte 31270-901, MG, Brazil

**Keywords:** SARS-CoV-2, diagnostic tests, meta-analysis, systematic review, sensitivity, specificity

## Abstract

In this paper, we present a systematic review and meta-analysis that aims to evaluate the reliability of coronavirus disease diagnostic tests in 2019 (COVID-19). This article seeks to describe the scientific discoveries made because of diagnostic tests conducted in recent years during the severe acute respiratory syndrome coronavirus 2 (SARS-CoV-2) pandemic. Between 2020 and 2021, searches for published papers on the COVID-19 diagnostic were made in the PubMed database. Ninety-nine scientific articles that satisfied the requirements were analyzed and included in the meta-analysis, and the specificity and sensitivity of the diagnostic accuracy were assessed. When compared to serological tests such as the enzyme-linked immunosorbent assay (ELISA), chemiluminescence immunoassay (CLIA), lateral flow immunoassay (LFIA), and chemiluminescent microparticle immunoassay (CMIA), molecular tests such as reverse transcription polymerase chain reaction (RT-PCR), reverse transcription loop-mediated isothermal amplification (RT-LAMP), and clustered regularly interspaced short palindromic repeats (CRISPR) performed better in terms of sensitivity and specificity. Additionally, the area under the curve restricted to the false-positive rates (AUC_FPR_) of 0.984 obtained by the antiviral neutralization bioassay (ANB) diagnostic test revealed significant potential for the identification of COVID-19. It has been established that the various diagnostic tests have been effectively adapted for the detection of SARS-CoV-2; nevertheless, their performance still must be enhanced to contain potential COVID-19 outbreaks, which will also help contain potential infectious agent outbreaks in the future.

## 1. Introduction

The end of 2019 saw the first mention of the coronavirus disease 2019 (COVID-19), which is brought on by the severe acute respiratory syndrome coronavirus 2 (SARS-CoV-2) [1]. Due to COVID-19’s extensive global spread, the World Health Organization (WHO) designated it a pandemic on 11 March 2020 [2]. The virus is spread through direct physical contact with an infected person, the expulsion of droplets and tiny particles during breathing, speaking, or coughing, or the deposition of saliva-containing droplets on surfaces and/or objects that are then touched [3,4]. By September 2022, the WHO estimated that there would have been 600 million confirmed cases of COVID-19 reported globally, resulting in more than 6 million fatalities [5]. Early detection of infection would enable the prevention of the rapid spread of the virus [6]. Similarly, the transmission of the virus by asymptomatic people is a serious concern for community spread [7], where a study showed that 35.1% of patients with COVID-19 did not present symptoms [8]. According to estimates, the average direct medical cost for a single symptomatic COVID-19 infection would be USD 3045, rising to USD 14,366 for each hospitalization [9], making COVID-19 one of the most serious global health disasters in history.

The association between the S protein of the virus and the host membrane receptor angiotensin-converting enzyme 2 (ACE2) is important to the pathogenesis of SARS-CoV-2 [10]. In addition to the respiratory system, COVID-19 also has an impact on the heart, blood vessels, liver, kidneys, lungs, and liver [11]. Many COVID-19 symptoms are fever, coughing, dyspnea, malaise, exhaustion, neurological symptoms, dermatological manifestations, anorexia, myalgia, sneezing, sore throat, rhinitis, goosebumps, headache, chest discomfort, and diarrhea [12,13]. Acute respiratory distress syndrome (ARDS), which is brought on by an uncontrolled inflammatory/immune system and cytokine storm [14,15], may be related to the mild symptoms that SARS-CoV-2 causes in some patients. However, these symptoms may also be related to an earlier onset of generalized infection in the lungs. COVID-19 infection has also been linked to several control tactics against the spread of COVID-19 in many nations initially centered on full or partial lockdowns, but in many countries, lockdowns were either the entire or a portion of the strategy. However, because of the sharp rise in the number of hospitalized infected patients, these interventions were not as successful [16]. The incidence of hospitalization and death has been reduced by some licensed vaccines, but vaccination coverage is still low [17,18] and many of them may not be effective against emerging viral variations [19]. Additional potential late complications include venous thromboembolism, arterial thrombosis, pulmonary fibrosis, cardiac thrombosis and inflammation, cerebrovascular accident, mental fog, dermatological complications, and general mood dysfunctions [20].

Control strategies against the spread of COVID-19 in many countries initially focused on complete or partial lockdowns, but in many countries, control strategies against the spread of COVID-19 initially focused on complete or partial lockdowns. Still, these measures were not as effective due to the rapid increase in the number of hospitalized infected people [16]. Several vaccines have been licensed to reduce the incidence of hospitalization and death; despite this, however, vaccination coverage remains insufficient [17,18] and there is a possibility that it may not be effective against newer virus variants [19]. The primary method for treating critically sick patients who require ventilator support in the intensive care unit has been symptomatic treatment rather than curative treatment [21]. Antiviral therapy, antibiotic therapy, and immunomodulatory therapy are further potential therapeutic approaches [22]. To quickly apply control measures to stop SARS-CoV-2 transmission by case identification, contact tracing, and isolation of positive cases [23,24], diagnostic technologies continue to be essential in the fight against COVID-19.

For individuals who got very ill at the start of the COVID-19 epidemic, there was no effective diagnostic test available. The patient’s clinical symptoms and exposure history were the major factors in making the diagnosis. Because the SARS-CoV-2 genome had been sequenced, WHO was able to create a real-time RT-PCR-based procedure for the molecular diagnosis of COVID-19, which allowed the creation of marketed diagnostic kits [25]. The current reference laboratory test is real-time RT-PCR, which is regarded as the “gold standard” due to its excellent sensitivity and specificity [26]. The expensive cost, the requirement for ribonucleic acid (RNA) extraction, the availability of specialized raw materials, and the relatively lengthy execution time make it difficult to apply on a broad scale, despite the high diagnostic accuracy [27,28]. Serological tests, on the other hand, are dependable, straightforward, and affordable methods that enable both direct and indirect detection of infections; nevertheless, they also identify the presence of antibodies as a sign of prior infection [29]. The identification of IgA, IgG, and IgM antibodies from the patient’s serum or plasma that are directed against the spike (S) and the nucleocapsid (N) proteins of SARS-CoV-2 is frequently done using serologic assays, including ELISA, CLIA, and LFIA as diagnostic tools [30]. Its use in acute phase diagnosis is constrained by the time of immunoglobulin production (from 4 days after the beginning of symptoms to 10–14 days). However, by combining serological tests with molecular tests, it may be possible to detect IgM and IgA that are quickly generated in response to infection, which could assist diagnosing COVID-19 and dramatically improving diagnostic sensitivity [31,32]. The diagnosis of active infection is made possible by COVID-19 antigen tests, which recognize SARS-CoV-2 virus proteins in a variety of substrates. They can be processed and viewed using a small handheld device or visually read. They are provided as single-use, quick antigen detection assays that flow laterally. Both procedures can be carried out outside of a lab and take 15 to 20 min to perform. These tests might be developed more quickly and applied more widely. These tests are often less sensitive than molecular assays but have the potential to be exceedingly accurate [33,34,35].

The current study intends to comprehensively examine and synthesize the literature that is currently available on the diagnostic efficacy of COVID-19 diagnostic tests. In this sense, between 2020 and 2021, a thorough evaluation of the literature was conducted. A meta-analysis based on the methods created and applied for the diagnosis of COVID-19 was performed to examine the findings. The following diagnostic methods were examined: RT-PCR (reverse transcription polymerase chain reaction), RT-LAMP (reverse transcriptase loop-mediated isothermal amplification), CRISPR, microarrays (MA), NGS, ELISA (enzyme-linked immunosorbent assay), antiviral neutralization bioassay (ANB), biosensors (BS), chemiluminescence immunoassay (CLIA), lateral flow immunoassay (FIA). Therefore, we anticipate that the gathered data will aid in identifying the most efficient diagnostic methods.

## 2. Materials and Methods

### 2.1. Search Strategy

This systematic review was based on the PRISMA (Preferred Reporting Items for Systematic Reviews and Meta-Analyses) technique [36]. This systematic review protocol has been registered on INPLASY [https://inplasy.com/inplasy-2022-9-0132 (accessed on 16 November 2022)]. The registration number is INPLASY202290132. The review has been elaborated according to PRISMA 2020 checklist (Table S1) [36].

The search was conducted in the PubMed database [https://pubmed.ncbi.nlm.nih.gov (accessed on 10 June 2022)] until 10 June 2022. The US NLM/National Center for Biotechnology Information developed and supports PubMed, one of the most used search engines for biomedical literature (NCBI) [37]. Using the MeSH term “COVID-19”, terms linked with the diagnosis of COVID-19 were sought out in the literature. The findings were displayed in a co-occurrence network map of MeSH terms in the VOSviewer software (version 1.6.18) [38]. To choose pertinent terms related to COVID-19 diagnostic methods, the clusters in the network map were examined. Additionally, a second search was created using each MeSH phrase found through the cluster analysis and related to the MeSH terms “sensitivity and specificity”, which are often thought of as a metric for assessing the diagnostic efficacy of a test [39], and the MeSH term “COVID-19”.

### 2.2. Search Strategy, Eligibility Criteria, and Data Extraction

Three processes went into selecting the studies for the meta-analysis. Only studies published between 2020 and 2021 and involving human subjects were included in the first stage of the identification stage, which also excluded duplicate articles, articles written in languages other than English, reviews, and meta-analyses. The titles and abstracts of the articles discovered using the search method were examined in the second stage of the screening stage. The entire studies with high relevance were retrieved and separated from the papers with titles or abstracts that lacked the necessary information to be taken into account during the eligibility stage. The diagnostic method, the quantity, variety, and clinical characteristics of the COVID-19 patients, as well as healthy controls, were all gathered from each chosen study. All studies that assessed diagnostic accuracy using sensitivity and specificity measures were included. Additionally, information about the geographical distribution, the number of studies associated by nation, and the frequency of diagnostic methods utilized were retrieved. Studies lacking information and having conflicting incomplete information about the COVID-19 diagnostic tests’ sensitivity and specificity were not included in the study.

### 2.3. Statistical Analysis

Results were entered into Microsoft Excel (version 10.0, Microsoft Corporation, Redmond, WA, USA) spreadsheets and analyzed in the R programming environment (version 4.2.1) using the package “mada” (version 0.5.11) https://cran.r-project.org/web/packages/mada/index.html (accessed on 24 October 2022); which employs a hierarchical model that accounts for within and between-study (heterogeneity) and the correlation between sensitivity and specificity [40]. Initially, the number of true negatives (TP), false negatives (FN), true positives (TP), and false positives (FP) were analyzed separately for each diagnostic technique; while the evaluation of sensitivity (Se) and specificity (Sp) made it possible to determine the diagnostic performance. Additionally, the positive likelihood ratio (LR+) expresses the ratio between the probability of expecting a positive test in a patient and the probability of expecting a positive test in a patient without the disease [41]; the negative likelihood ratio (LR−), which expresses the probability that a patient will test negative among people with the disease and the probability that a patient will test negative among people without disease; and the diagnostic likelihood ratio (DOR), which is the odds ratio of the positivity of a diagnostic test result in the diseased population relative to the non-diseased population [42]; and the 95% confidence interval (CI) were determined. Summary receiver operating characteristic (sROC) curves were fitted, according to the parameters of the “Reitsma” model of the “mada” package and were used to compare the diagnostic accuracy of CD diagnostic techniques [43]. The confidence level for all calculations was set to 95%, using a continuity correction of 0.5 if pertinent.

## 3. Results

### 3.1. Data Sources and Study Selection

**Figure 1:**
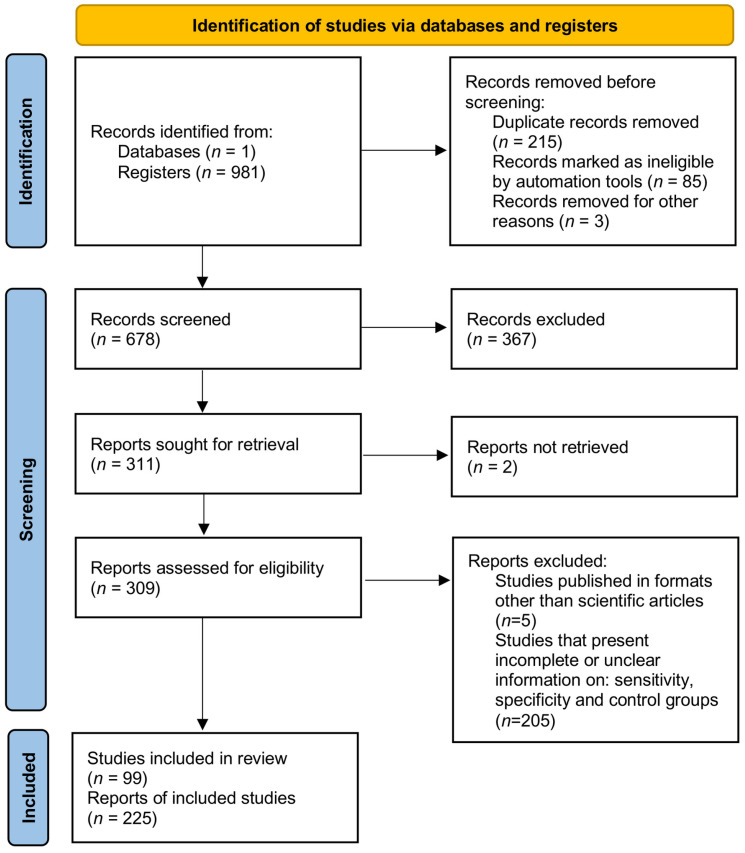
A systematic review and meta-analysis flow diagram of the study selection process.

**Figure 2:**
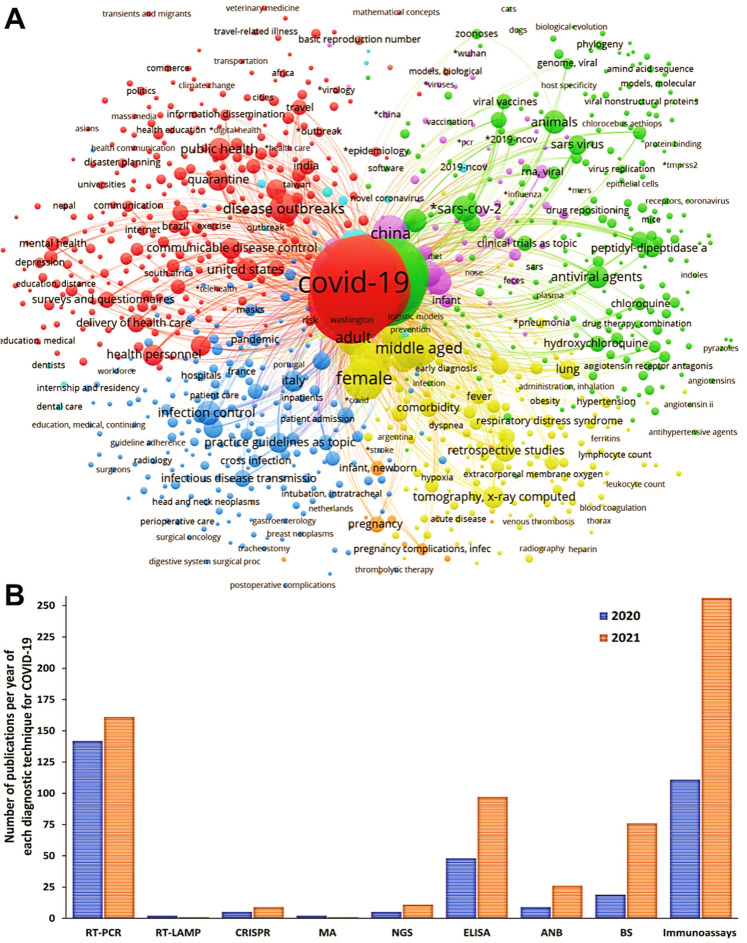
Selected articles using the PubMed database for the different diagnostic techniques using MeSH terms. (**A**) Network map built by VOSviewer based on the co-occurrence of MeSH terms. (**B**) Number of articles found with the search for each diagnostic test, considered from cluster analysis.

In this study, a systematic review followed by meta-analysis was performed to measure the accuracy of diagnostic tests for COVID-19. A flowchart of the study strategy was prepared and presented (Figure 1). To this end, a search was made in the PubMed database with the MeSH terms “COVID-19”, and a co-occurrence network map of MeSH terms was developed; Through the search, 981 scientific articles were obtained between the years 2020 and 2021. The minimum number of occurrences of keywords was set at a value of five, and a network map with 2.518 keywords was generated (Figure 2A). The formation of five main clusters was found in the analysis of the network map, in the cluster related to diagnostic techniques (purple color) terms such as “Reverse Transcription–Polymerase Chain Reaction”, “Reverse Transcriptase Loop-Mediated Isothermal Amplification”, “Clustered Regularly Interspaced Short Palindromic Repeats”, “Microarrays”, “Next-Generation Sequencing”, “Enzyme-Linked Immunosorbent Assay”, “Antiviral Neutralization Bioassay”, “Biosensor”, and “Immunoassay”. In addition, terms such as “COVID-19”, “SARS-CoV-2”, “adult”, China”, “disease outbreaks middle-aged”, and “female” were common denominators (Figure 2A).

A second search was performed in the PubMed database with the terms found in the first analysis. The new terms were associated with the terms “COVID-19” and “Sensitivity and Specificity”; generating the new search strings: (COVID-19[MeSH Terms]) AND (sensitivity and specificity[MeSH Terms]) AND (RT-PCR[MeSH Terms]) for RT-PCR; (COVID-19[MeSH Terms]) AND (sensitivity and specificity[MeSH Terms]) AND (RT-LAMP assay[MeSH Terms]) for RT-LAMP; (COVID-19[MeSH Terms]) AND (sensitivity and specificity[MeSH Terms]) AND (CRISPR[MeSH Terms]) for CRISPR; (COVID-19[MeSH Terms]) AND (sensitivity and specificity[MeSH Terms]) AND (Microarray Analysis[MeSH Terms]) for MA; (COVID-19[MeSH Terms]) AND (sensitivity and specificity[MeSH Terms]) AND (Next generation sequencing[MeSH Terms]) for NGS; (COVID-19[MeSH Terms]) AND (sensitivity and specificity[MeSH Terms]) AND (ELISA[MeSH Terms]) for ELISA; (COVID-19[MeSH Terms]) AND (sensitivity and specificity[MeSH Terms]) AND (Neutralization Tests[MeSH Terms]) for ANB; (COVID-19[MeSH Terms]) AND (sensitivity and specificity[MeSH Terms]) AND (Biosensing Technique[MeSH Terms]) for BS; and (COVID-19[MeSH Terms]) AND (sensitivity and specificity[MeSH Terms]) AND (Immunoassay[MeSH Terms]) for immunoassays.

**Figure 3:**
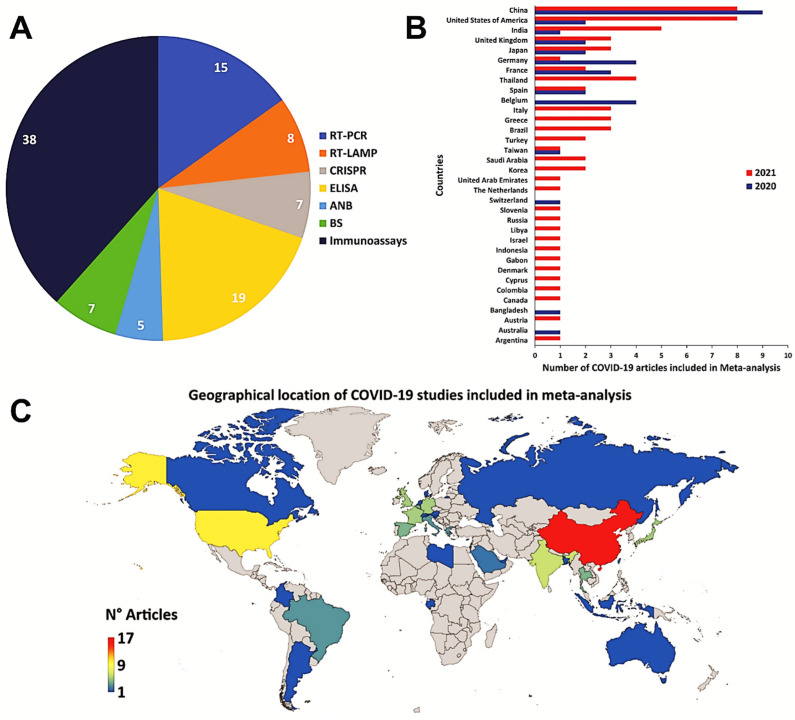
The geographical location of COVID-19 studies. (**A**) The pie chart shows the type of diagnostic tests used in the COVID-19 studies for the meta-analysis. (**B**) The bar graph shows the number of COVID-19 studies, included in the meta-analysis, carried out by different countries. (**C**) Demographic representation of COVID-19 studies, included in the meta-analysis, worldwide (lower-blue to upper-red numbers).

The number of studies chosen for RT-PCR, RT-LAMP, CRISPR, MA, NGS, ELISA, ANB, BS, and immunoassays were: 303, 3, 14, 3, 16, 145, 35, 95, and 367, respectively (Figure 2B). The three-step eligibility criterion allowed 303, 369, and 215 articles to be excluded, from the criteria for identification, screening, and eligibility, respectively. Therefore, 99 articles were selected for meta-analysis (Figure 3). It is observed that in most studies, the diagnostic techniques used were immunoassays (CLIA, LFIA, CMIA, ECLIA, and FIA) (Figure 3A). Additionally, China, the United States of America, and India are the countries that have carried out a higher number of studies related to diagnostic tests for COVID-19 (Figure 3B,C).

### 3.2. Meta-Analysis of the Diagnostic Techniques for COVID-19

#### 3.2.1. Reverse Transcription–Polymerase Chain Reaction

**Figure 4:**
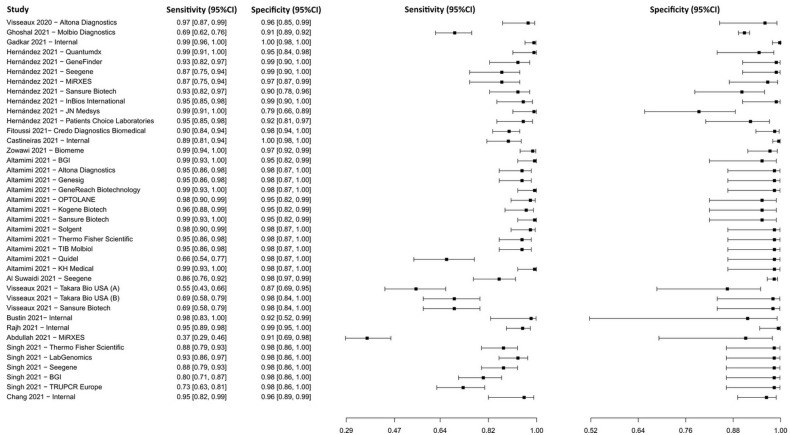
Data analysis and paired forest plot of the sensitivity and specificity of the reverse transcription–polymerase chain reaction (RT-PCR) in the diagnosis of COVID-19. Sensitivity and specificity are reported as mean (95% confidence limits). The forest plot represents the estimated sensitivity and specificity (black squares) and their 95% confidence limits (horizontal black line) [44,45,46,47,48,49,50,51,52,53,54,55,56,57,58].

Fifteen studies based on the RT-PCR technique were selected [44,45,46,47,48,49,50,51,52,53,54,55,56,57,58], in which a total of 6902 subjects were studied. Sensitivity ranged from 36.8% to 99.2%, with a median of 94.5%, 95%CI (85.1, 98.0), while the test for equality of sensitivities presented a χ^2^ = 577.02, df = 38, *p*-value = 2 × 10^−16^. Specificity ranged from 79.3 to 99.8%, with a median of 98.4%, 95%CI (86.7, 99.8); the test for equality of specificities showed χ^2^ = 142.18, df = 38, *p*-value = 5.96 × 10^−14^. A negative correlation between sensitivities and false positive rates is shown r = –0.188, 95%CI (–0.476, 0.135). Additionally, results regarding LR+ {median 45.00, 95%CI (4.06, 563.27)}, LR− {median 0.06, 95%CI (0.02, 0.18)}, and DOR {median 609.00, 95%CI (54.53, 8485.95)}. The analyzed diagnostic performance is summarized in Figure 4 and Supplementary Figure S1.

#### 3.2.2. Reverse Transcriptase Loop-Mediated Isothermal Amplification

Seven studies were selected using the RT-LAMP technique [59,60,61,62,63,64,65]. A total of 1806 subjects were studied. Sensitivity ranged from 74.7 to 98.8%, with a median of 91.9%, 95%CI (80.0, 97.0); while the test for equality of sensitivities showed: χ^2^ = 30.09, df = 7, *p*-value = 9.12 × 10^−05^. Specificity ranged from 88.1 to 99.6%, with a median of 98.8%, 95%CI (90.0, 100.0); while the test for equality of specificities presented χ^2^ = 34.71, df = 7, *p*-value = 1.27 × 10^−05^.

**Figure 5:**
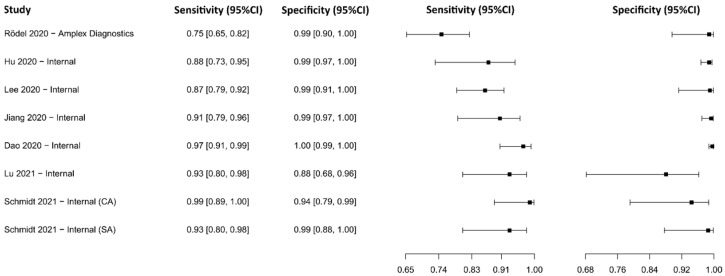
Data analysis and paired forest plot of the sensitivity and specificity of the reverse transcriptase loop-mediated isothermal amplification (RT-LAMP) in the diagnosis of COVID-19. Sensitivity and specificity are reported as mean (95% confidence limits). The forest plot represents the estimated sensitivity and specificity (black squares) and their 95% confidence limits (horizontal black line) [59,60,61,62,63,64,65].

The correlation between sensitivities and false positive rates was analyzed and a negative result is shown r = 0.313, 95%CI (−0.502, 0.834). In addition, results regarding LR+ {median 69.51, 95%CI (4.88, 755.24)}, LR− {median 0.09, 95%CI (0.03, 0.23)}, and DOR {median 801.74, 95%CI (48.36, 10,044.33)} are displayed. The analyzed diagnostic performance is summarized in Figure 5 and Supplementary Figure S2.

#### 3.2.3. Clustered Regularly Interspaced Short Palindromic Repeats

**Figure 6:**
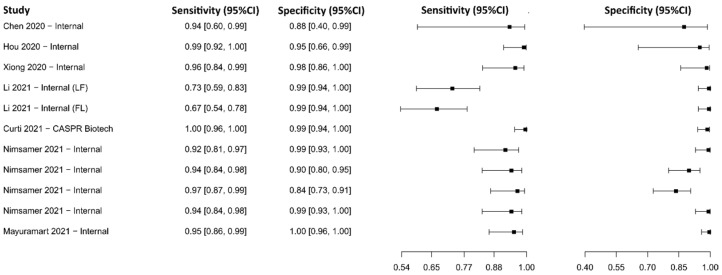
Data analysis and paired forest plot of the sensitivity and specificity of the clustered regularly interspaced short palindromic repeats (CRISPR) in the diagnosis of COVID-19. Sensitivity and specificity are reported as mean (95% confidence limits). The forest plot represents the estimated sensitivity and specificity (black squares) and their 95% confidence limits (horizontal black line) [66,67,68,69,70,71,72].

The analysis identified 14 published studies that used CRISPR as a diagnostic tool. After analysis, only seven studies [66,67,68,69,70,71,72] were selected. A total of 1201 subjects were studied. Sensitivity ranged from 67.0 to 99.5%, with a median of 94.4%, and 95%CI (84.0, 99.0). Test for equality of sensitivities analysis showed: χ^2^ = 80.26, df = 10, *p*-value = 4.47 × 10^−13^. Specificity ranged from 83.6 to 99.6%, with a median of 98.6%, 95%CI (93.0, 100.0); while the test for equality of specificities: χ^2^ = 55.37, df = 10, *p*-value = 2.69 × 10^−08^. Additionally, a negative correlation between sensitivities and false positive rates is shown r = 0.328, 95%CI (–0.339, 0.775). In addition, results regarding LR+ {median 70.33, 95%CI (6.80, 898.96)}, LR− {median 0.06, 95%CI (0.01, 0.20)}, and DOR {median 1357, 95%CI (37.27, 29,912.15)} are displayed. The diagnostic performance of the selected studies is summarized in Figure 6 and Supplementary Figure S3.

#### 3.2.4. Enzyme-Linked Immunosorbent Assay for IgG

**Figure 7:**
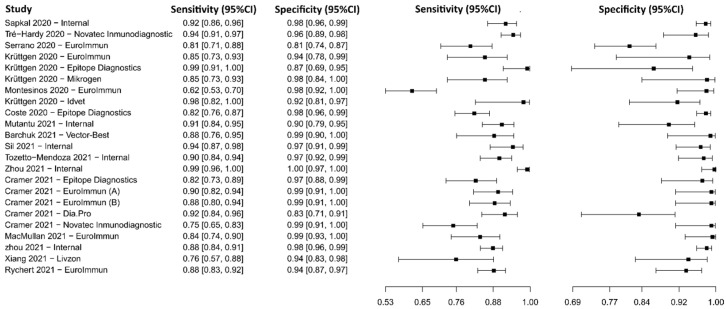
Data analysis and paired forest plot of the sensitivity and specificity of the enzyme-linked immunosorbent assay (ELISA) for IgG in the diagnosis of COVID-19. Sensitivity and specificity are reported as mean (95% confidence limits). The forest plot represents the estimated sensitivity and specificity (black squares) and their 95% confidence limits (horizontal black line) [73,74,75,76,77,78,79,80,81,82,83,84,85,86,87,88].

Sixteen studies were selected using IgG-detecting ELISA as a diagnostic technique [73,74,75,76,77,78,79,80,81,82,83,84,85,86,87,88]. A total of 9221 subjects were studied. Sensitivity ranged from 61.6 to 99.0%, with a median of 88.3%, 95%CI (80.3, 93.5), while the test for equality of sensitivities presented a χ^2^ = 151.80, df = 22, *p*-value = 2 × 10^−16^. Specificity ranged from 81.3 to 99.7%, with a median of 97.4%, 95%CI (89.6, 98.9); the test for equality of specificities showed χ^2^ = 135.69, df = 22, *p*-value = 2 × 10^−16^. A negative correlation between sensitivities and false positive rates is shown r = 0.166, 95%CI (–0.264, 0.541). In addition, the results regarding LR+ {median 29.99, 95%CI (5.78, 80.78)}, LR− {median 0.12, 95%CI (0.06, 0.23)}, and DOR {median 333.00, 95%CI (37.03, 1288.51)}. The analyzed diagnostic performance is summarized in Figure 7 and Supplementary Figure S4.

#### 3.2.5. Enzyme-Linked Immunosorbent Assay for IgM

**Figure 8:**
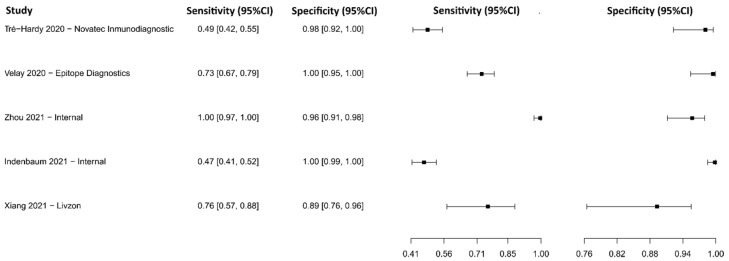
Data analysis and paired forest plot of the sensitivity and specificity of the enzyme-linked immunosorbent assay (ELISA) for IgM in the diagnosis of COVID-19. Sensitivity and specificity are reported as mean (95% confidence limits). The forest plot represents the estimated sensitivity and specificity (black squares) and their 95% confidence limits (horizontal black line) [74,84,87,89,90].

Five studies were selected using IgM-detecting ELISA as a diagnostic tool [74,84,87,89,90], in which a total of 1585 subjects were studied. Sensitivity ranged from 46.9% to 99.7%, with a median of 73.1%, 95%CI (57.3, 78.8), while the test for equality of sensitivities presented a χ^2^ = 150.59, df = 4, *p*-value = 2 × 10^−16^. Specificity ranged from 89.3 to 99.8%, with a median of 98.1%, 95%CI (92.3, 99.6); the test for equality of specificities showed χ^2^ = 28,12, df = 4, *p*-value = 1.18 × 10^−05^. A negative correlation between sensitivities and false positive rates is shown r = 0.446, 95%CI (–0.719, 0.953). Additionally, results regarding LR+ {median 25.90, 95%CI (9.29, 127.19)}, LR− {median 0.27, 95%CI (0.21, 0.53)}, and DOR {median 546.65, 95%CI (33.36, 8956.27)} are displayed. The analyzed diagnostic performance is summarized in Figure 8 and Supplementary Figure S5.

#### 3.2.6. Enzyme-Linked Immunosorbent Assay for IgA

Five studies were selected using IgA-detecting ELISA as a diagnostic technique [74,75,77,89,90]. A total of 1632 subjects were studied. Sensitivity ranged from 79.8 to 92.4%, with a median of 83.7%, 95%CI (77.9, 88.8); while the test for equality of sensitivities showed: χ^2^ = 13.71, df = 4, *p*-value = 8.25 × 10^−03^. Specificity ranged from 85.6 to 99.6%, with a median of 98.0%, 95%CI (92.3, 99.1); while the test for equality of specificities presented χ^2^ = 34.46, df = 4, *p*-value = 6.00 × 10^−07^.

**Figure 9:**
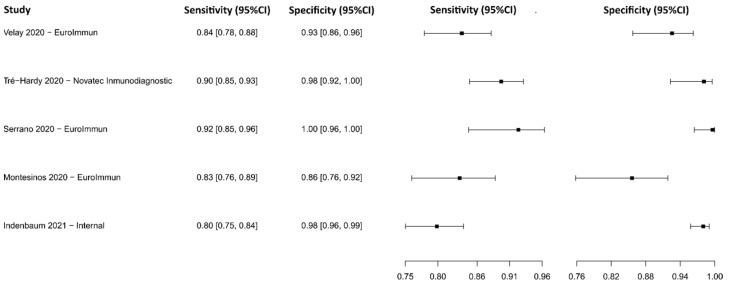
Data analysis and paired forest plot of the sensitivity and specificity of the enzyme-linked immunosorbent assay (ELISA) for IgA in the diagnosis of COVID-19. Sensitivity and specificity are reported as mean (95% confidence limits). The forest plot represents the estimated sensitivity and specificity (black squares) and their 95% confidence limits (horizontal black line) [74,75,77,89,90].

The correlation between sensitivities and false positive rates was analyzed and a negative result is shown r = −0.448, 95%CI (−0.953, 0.718). In addition, the results regarding LR+ {median 39.92, 95%CI (9.80, 85.62)}, LR− {median 0.18, 95%CI (0.13, 0.24)}, and DOR {median 194.04, 95%CI (85.07, 442.62)} are displayed. The analyzed diagnostic performances are summarized in Figure 9 and Supplementary Figure S6.

#### 3.2.7. Antiviral Neutralization Bioassay

**Figure 10:**
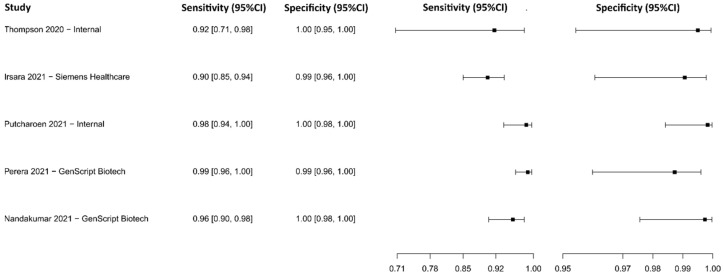
Data analysis and paired forest plot of the sensitivity and specificity of the antiviral neutralization bioassay (ANB) in the diagnosis of COVID-19. Sensitivity and specificity are reported as mean (95% confidence limits). The forest plot represents the estimated sensitivity and specificity (black squares) and their 95% confidence limits (horizontal black line) [91,92,93,94,95].

The analysis identified 40 studies that used ANB as a diagnostic technique for COVID-19. After analysis, only five studies [91,92,93,94,95] were selected. A total of 1567 subjects were studied. Sensitivity ranged from 90.2 to 98.8%, with a median of 95.6%, and 95%CI (90.0, 98.0). Test for equality of sensitivities analysis showed: χ^2^ = 19.18, df = 4, *p*-value = 7.23 × 10^−04^. The specificity of the studies ranged from 98.7 to 99.8%, with a median of 99.5%, 95%CI (96.0, 100.0); while the test for equality of specificities: χ^2^ = 3.21, df = 4, *p*-value = 0.52. Additionally, a negative correlation between sensitivities and false positive rates is shown r = −0.055, 95%CI (–0.894, 0.869). In addition, results regarding LR+ {median 185.17, 95%CI (22.71, 2950.41)}, LR− {median 0.04, 95%CI (0.02, 0.10)}, and DOR {median 6332.92, 95%CI (458.41, 56,606.18)} are displayed. The diagnostic performance of the selected studies is summarized in Figure 10 and Supplementary Figure S7.

#### 3.2.8. Biosensors

**Figure 11:**
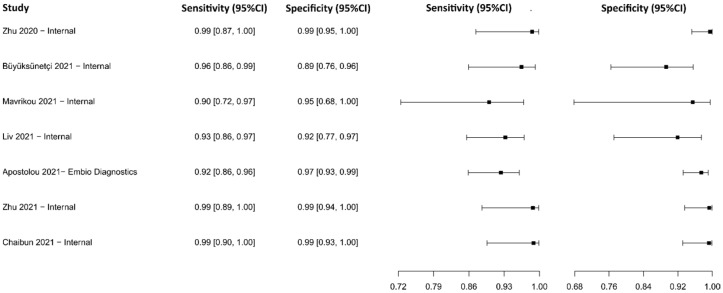
Data analysis and paired forest plot of the sensitivity and specificity of the biosensors (BS) in the diagnosis of COVID-19. Sensitivity and specificity are reported as mean (95% confidence limits). The forest plot represents the estimated sensitivity and specificity (black squares) and their 95% confidence limits (horizontal black line) [96,97,98,99,100,101,102].

Seven studies were selected using BS as a diagnostic technique [96,97,98,99,100,101,102]. A total of 814 subjects were studied. Sensitivity ranged from 90.0 to 98.8%, with a median of 96.4%, and CI of 95% (85.9, 99.2), while the test for equality of sensitivities presented a χ^2^ = 6.63, df = 6, *p*-value = 0.35. Specificity ranged from 89.3 to 99.5%, with a median of 97.4%, 95%CI (93.1, 99.5); the test for equality of specificities showed χ^2^ = 17.31, df = 6, *p*-value = 0.01. A negative correlation between sensitivities and false positive rates is shown r = 0.385, 95%CI (–0.882, 0.518). In addition, results regarding LR+ {median 36.15, 95%CI (8.24, 297.95)}, LR− {median 0.04, 95%CI (0.01, 0.19)}, and DOR {median 459.96, 95%CI (129.02, 4295.06)} are displayed. The analyzed diagnostic performance is summarized in Figure 11 and Supplementary Figure S8.

#### 3.2.9. Chemiluminescence Immunoassay for IgG

**Figure 12:**
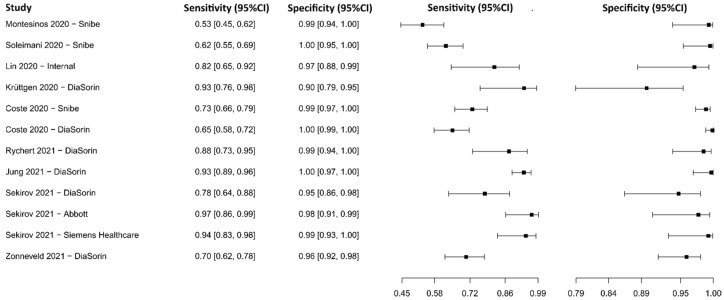
Data analysis and paired forest plot of the sensitivity and specificity of the chemiluminescence immunoassay (CLIA) for IgG in the diagnosis of COVID-19. Sensitivity and specificity are reported as mean (95% confidence limits). The forest plot represents the estimated sensitivity and specificity (black squares) and their 95% confidence limits (horizontal black line) [76,77,79,88,103,104,105,106,107].

Eight studies were selected using CLIA as an IgG detection technique [76,77,79,88,103,104,105,106,107], in which a total of 2859 subjects were studied. Sensitivity ranged from 53.1% to 96.5%, with a median of 79.8%, 95%CI (65.3, 89.7), while the test for equality of sensitivities presented a χ^2^ = 111.96, df = 11, *p*-value = 2 × 10^−16^. Specificity ranged from 89.8 to 99.9%, with a median of 98.7%, 95%CI (93.5, 99.6); the test for equality of specificities showed χ^2^ = 47.84, df = 11, *p*-value = 1.53 × 10^−06^. A negative correlation between sensitivities and false positive rates is shown r = 0.319, 95%CI (–0.312, 0.755). Additionally, results regarding LR+ {median 62.27, 95%CI (7.99, 248.31)}, LR− {median 0.21, 95%CI (0.11, 0.38)}, and DOR {median 286.73, 95%CI (46.74, 2894.79)} are displayed. The analyzed diagnostic performance is summarized in Figure 12 and Supplementary Figure S9.

#### 3.2.10. Chemiluminescence Immunoassay for IgM

Five studies were selected using CLIA as an IgM detection technique [77,79,103,104,106]. A total of 1240 subjects were studied. Sensitivity ranged from 58.7 to 89.5%, with a median of 61.7%, 95%CI (53.0, 77.0); while the test for equality of sensitivities showed: χ^2^ = 18.52, df = 4, *p*-value = 9.76 × 10^−04^. Specificity ranged from 91.3 to 99.5%, with a median of 99.2%, 95%CI (94.0, 100.0); while the test for equality of specificities presented χ^2^ = 13.50, df = 4, *p*-value = 9.06 × 10^−03^.

**Figure 13:**
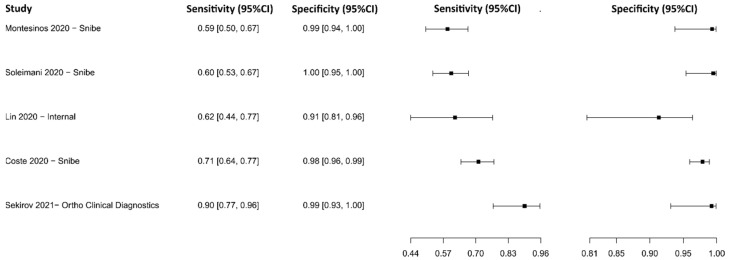
Data analysis and paired forest plot of the sensitivity and specificity of the chemiluminescence immunoassay (CLIA) for IgM in the diagnosis of COVID-19. Sensitivity and specificity are reported as mean (95% confidence limits). The forest plot represents the estimated sensitivity and specificity (black squares) and their 95% confidence limits (horizontal black line) [77,79,103,104,106].

The correlation between sensitivities and false positive rates was analyzed and a negative result is shown r = −0.252, 95%CI (−0.928, 0.810). In addition, results regarding LR+ {median 85.65, 95%CI (7.46, 1361.54)}, LR− {median 0.40, 95%CI (0.27, 0.48)}, and DOR {median 250.76, 95%CI (18.56, 3396.17)} are displayed. The analyzed diagnostic performances are summarized in Figure 13 and Supplementary Figure S10.

#### 3.2.11. Chemiluminescence Immunoassay for IgM-IgG

**Figure 14:**
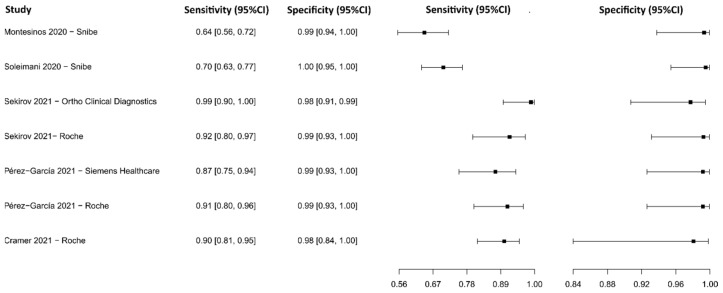
Data analysis and paired forest plot of the sensitivity and specificity of the chemiluminescence immunoassay (CLIA) for IgM-IgG in the diagnosis of COVID-19. Sensitivity and specificity are reported as mean (95% confidence limits). The forest plot represents the estimated sensitivity and specificity (black squares) and their 95% confidence limits (horizontal black line) [77,85,103,106,108].

The analysis identified 28 published studies that used CLIA to detect IgM-IgG antibodies for COVID-19. After analysis, only five studies [77,85,103,106,108] were selected. A total of 1008 subjects were studied. Sensitivity ranged from 64.2 to 98.8%, with a median of 90.1%, and 95%CI (80.0, 95.0). Test for equality of sensitivities analysis showed: χ^2^ = 51.25, df = 6, *p*-value = 2.64 × 10^−09^. The specificity of the studies ranged from 97.7 to 99.5%, with a median of 99.2%, 95%CI (93.0, 100.0); while the test for equality of specificities: χ^2^ = 1.73, df = 6, *p*-value = 0.94. Additionally, a negative correlation between sensitivities and false positive rates is shown r = 0.635, 95%CI (–0.226, 0.939). In addition, results regarding LR+ {median 106.45, 95%CI (7.03, 1685.99)}, LR− {median 0.10, 95%CI (0.05, 0.22)}, and DOR {median 828.39, 95%CI (45.47, 15,090.85)} are displayed. The diagnostic performance of the selected studies is summarized in Figure 14 and Supplementary Figure S11.

#### 3.2.12. Lateral Flow Immunoassay for IgG

**Figure 15:**
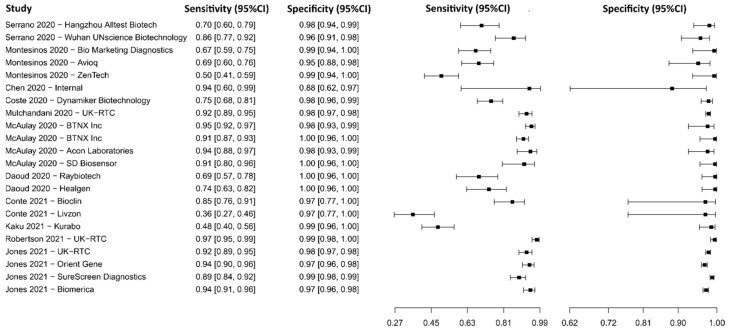
Data analysis and paired forest plot of the sensitivity and specificity of the lateral flow immunoassay (LFIA) for IgG in the diagnosis of COVID-19. Sensitivity and specificity are reported as mean (95% confidence limits). The forest plot represents the estimated sensitivity and specificity (black squares) and their 95% confidence limits (horizontal black line) [75,77,79,109,110,111,112,113,114,115,116].

Eleven studies were selected using LFIA as an IgG detection technique [75,77,79,109,110,111,112,113,114,115,116]. A total of 15,935 subjects were studied. Sensitivity ranged from 35.9 to 97.4%, with a median of 87.3%, 95%CI (76.5, 91.9), while the test for equality of sensitivities presented a χ^2^ = 666.12, df = 21, *p*-value = 2 × 10^−16^. Specificity ranged from 88.5 to 99.6%, with a median of 97.9%, 95%CI (95.5, 99.3); the test for equality of specificities showed χ^2^ = 48.01, df = 21, *p*-value = 6.85 × 10^−04^. A negative correlation between sensitivities and false positive rates is shown r = 0.175, 95%CI (–0.267, 0.555). In addition, results regarding LR+ {median 40.07, 95%CI (11.54, 131.19)}, LR− {median 0.13, 95%CI (0.08, 0.25)}, and DOR {median 502.97, 95%CI (56.53, 1796.41)} are displayed. The analyzed diagnostic performance is summarized in Figure 15 and Supplementary Figure S12.

#### 3.2.13. Lateral Flow Immunoassay for IgM

**Figure 16:**
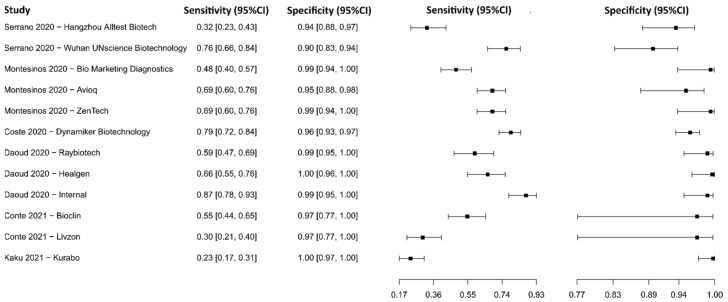
Data analysis and paired forest plot of the sensitivity and specificity of the lateral flow immunoassay (LFIA) for IgM in the diagnosis of COVID-19. Sensitivity and specificity are reported as mean (95% confidence limits). The forest plot represents the estimated sensitivity and specificity (black squares) and their 95% confidence limits (horizontal black line) [75,77,79,111,112,115].

Six studies were selected using LFIA as an IgM detection technique [75,77,79,111,112,115], in which a total of 2704 subjects were studied. Sensitivity ranged from 23.3% to 87.2%, with a median of 62.4%, 95%CI (51.1, 72.5), while the test for equality of sensitivities presented a χ^2^ = 208.82, df = 11, *p*-value = 2 × 10^−16^. Specificity ranged from 89.7 to 99.7%, with a median of 98.0%, 95%CI (93.7, 99.7); the test for equality of specificities showed χ^2^ = 40.45, df = 11, *p*-value = 3.00 × 10^−05^. A negative correlation between sensitivities and false positive rates is shown r = 0.153, 95%CI (–0.461, 0.669). Additionally, results regarding LR+ {median 33.70, 95%CI (5.07, 263.29)}, LR− {median 0.38, 95%CI (0.29, 0.51)}, and DOR {median 96.36, 95%CI (13.50, 651.54)} are displayed. The analyzed diagnostic performance is summarized in Figure 16 and Supplementary Figure S13.

#### 3.2.14. Lateral Flow Immunoassay for IgM-IgG

**Figure 17:**
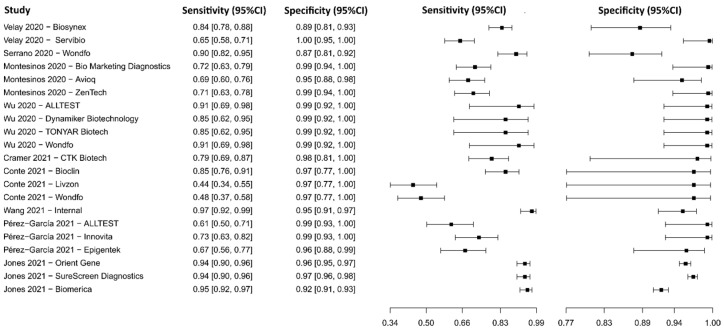
Data analysis and paired forest plot of the sensitivity and specificity of the lateral flow immunoassay (LFIA) for IgM-IgG in the diagnosis of COVID-19. Sensitivity and specificity are reported as mean (95% confidence limits). The forest plot represents the estimated sensitivity and specificity (black squares) and their 95% confidence limits (horizontal black line) [75,77,85,90,108,109,115,117,118].

Nine studies were selected using LFIA as an IgM-IgG detection technique [75,77,85,90,108,109,115,117,118]. A total of 9629 subjects were studied. Sensitivity ranged from 44.1 to 97.0%, with a median of 83.7%, 95%CI (63.4, 88.2); while the test for equality of sensitivities showed: χ^2^ = 339.59, df = 20, *p*-value = 2 × 10^−16^. Specificity of the studies ranged from 87.4 to 99.5%, with a median of 97.1%, 95%CI (92.4, 99.7); while the test for equality of specificities presented χ^2^ = 107.85, df = 20, *p*-value = 4.83 × 10^−14^. The correlation between sensitivities and false positive rates was analyzed and a negative result is shown r = 0.279, 95%CI (−0.173, 0.635). In addition, results regarding LR+ {median 30.97, 95%CI (6.32, 445.62)}, LR− {median 0.18, 95%CI (0.13, 0.39)}, and DOR {median 334.86, 95%CI (22.18, 2704.72)} are displayed. The analyzed diagnostic performances are summarized in Figure 17 and Supplementary Figure S14.

#### 3.2.15. Lateral Flow Immunoassay for N Protein

**Figure 18:**
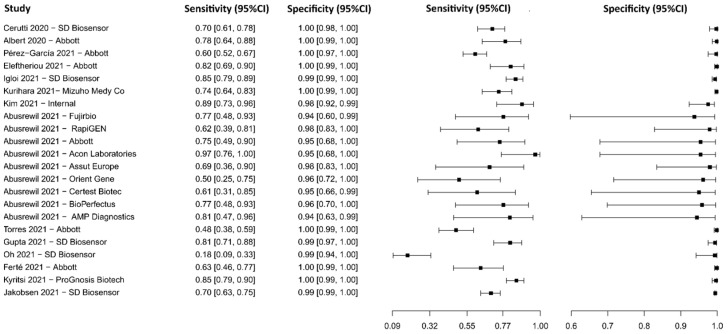
Data analysis and paired forest plot of the sensitivity and specificity of the lateral flow immunoassay (LFIA) for N-protein in the diagnosis of COVID-19. Sensitivity and specificity are reported as mean (95% confidence limits). The forest plot represents the estimated sensitivity and specificity (black squares) and their 95% confidence limits (horizontal black line) [119,120,121,122,123,124,125,126,127,128,129,130,131,132].

Fourteen studies were selected using LFIA as an N protein detection technique [119,120,121,122,123,124,125,126,127,128,129,130,131,132]. A total of 11,750 subjects were studied. Sensitivity ranged from 18.3 to 96.9%, with a median of 74.7%, 95%CI (50.7, 88.3), while the test for equality of sensitivities presented a χ^2^ = 145.62, df = 21, *p*-value = 2 × 10^−16^. Specificity ranged from 93.8 to 99.9%, with a median of 99.4%, 95%CI (95.9, 99.8); the test for equality of specificities showed χ^2^ = 56.33, df = 21, *p*-value = 4.51 × 10^−05^. A negative correlation between sensitivities and false positive rates is shown r = 0.193, 95%CI (–0.249, 0.568). In addition, results regarding LR+ {median 85.90, 95%CI (12.25, 482.92)}, LR− {median 0.26, 95%CI (0.12, 0.54)}, and DOR {median 501.51, 95%CI (46.03, 2611.02)} are displayed. The analyzed diagnostic performance is summarized in Figure 18 and Supplementary Figure S15.

#### 3.2.16. Chemiluminescent Microparticle Immunoassay

**Figure 19:**
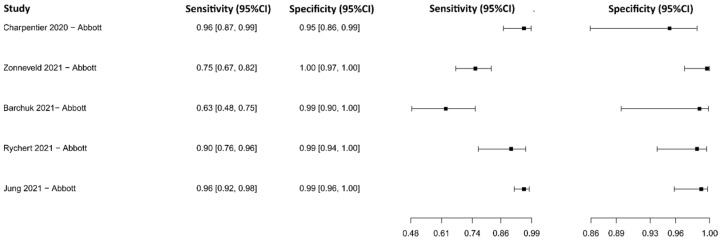
Data analysis and paired forest plot of the sensitivity and specificity of the chemiluminescent microparticle immunoassay (CMIA) in the diagnosis of COVID-19. Sensitivity and specificity are reported as mean (95% confidence limits). The forest plot represents the estimated sensitivity and specificity (black squares) and their 95% confidence limits (horizontal black line) [81,88,105,107,133].

The analysis identified 13 published studies that used CMIA as a diagnostic technique for COVID-19. After analysis, only five studies [81,88,105,107,133] were selected. A total of 939 subjects were studied. Sensitivity ranged from 62.8 to 95.7%, with a median of 90.3%, and 95%CI (76.4, 96.4). Test for equality of sensitivities analysis showed: χ^2^ = 51.58, df = 4, *p*-value = 1.69 × 10^−10^. Specificity ranged from 95.3 to 99.7%, with a median of 98.8%, 95%CI (93.8, 99.8); while the test for equality of specificities: χ^2^ = 6.26, df = 4, *p*-value = 0.18. Additionally, a negative correlation between sensitivities and false positive rates is shown r = 0.487, 95%CI (–0.693, 0.958). In addition, results regarding LR+ {median 60.79, 95%CI (12.37, 476.78)}, LR− {median 0.10, 95%CI (0.04, 0.27)}, and DOR {median 615.95, 95%CI (74.57, 4343.60)} are displayed. The diagnostic performance of the selected studies is summarized in Figure 19 and Supplementary Figure S16.

#### 3.2.17. Fluorescence Immunoassay

**Figure 20:**
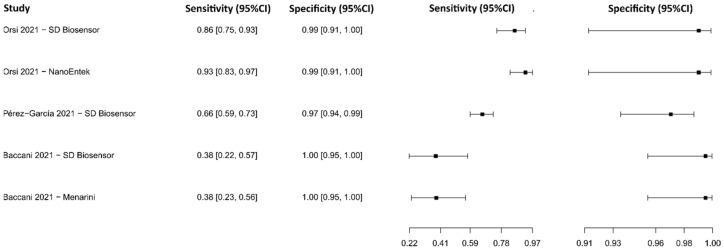
Data analysis and paired forest plot of the sensitivity and specificity of the fluorescence immunoassay (FIA) in the diagnosis of COVID-19. Sensitivity and specificity are reported as mean (95% confidence limits). The forest plot represents the estimated sensitivity and specificity (black squares) and their 95% confidence limits (horizontal black line) [121,134,135].

Three studies were selected using the FIA technique [121,134,135], in which a total of 829 subjects were studied. Sensitivity ranged from 38.0% to 92.6%, with a median of 64.4%, 95%CI (59.0, 73.0), while the test for equality of sensitivities presented a χ^2^ = 49.92, df = 4, *p*-value = 3.75 × 10^−10^. Specificity ranged from 97.1 to 99.5%, with a median of 99.0%, 95%CI (93.5, 99.9); the test for equality of specificities showed χ^2^ = 4.12, df = 4, *p*-value = 0.39. A negative correlation between sensitivities and false positive rates is shown r = 0.282, 95%CI (–0.799, 0.932). Additionally, results regarding LR+ {median 77.43, 95%CI (5.56, 1275.84)}, LR− {median 0.35, 95%CI (0.28, 0.43)}, and DOR {median 124.95, 95%CI (26.33, 2224.82)} are displayed. The analyzed diagnostic performance is summarized in Figure 20 and Supplementary Figure S17.

#### 3.2.18. Other Techniques

Regarding the NGS, MA, ELISA for IgG-IgM-IgA, ELISA for IgG-IgM/IgG-IgA, CLIA for IgG-IgM-IgA, CLIA for N protein, LFIA for S protein and ECLIA diagnostic techniques, one [89], zero, four [74,81,136,137], two [77,108], one [137], four [135,138,139,140], one [126], and three [105,107,111] studies were selected, respectively. According to the established criteria, at least five studies were needed for the analysis with a value of *p* < 0.05. So, no analysis was developed regarding these diagnostic techniques.

#### 3.2.19. Summary ROC Curves (sROC)

**Figure 21:**
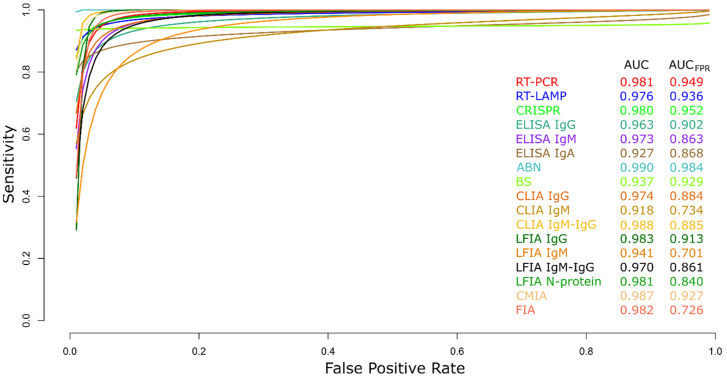
Meta-analysis of diagnostic test accuracy analysis. Summary receiver operating curve (sROC) plot of false positive rate and sensitivity. Comparison between RT-PCR, RT-LAMP, CRISPR, ELISA IgG, ELISA IgM, ELISA IgA, ABN, BS, CLIA IgG, CLIA IgM, CLIA IgM-IgG, LFIA IgG, LFIA IgM, LFIA IgM-IgG, LFIA N protein, CMIA, and FIA methods in the diagnosis of COVID-19.

Comparison of the diagnostic techniques data for COVID-19 (RT-PCR, RT-LAMP, CRISPR, ELISA IgG, ELISA IgM, ELISA IgA, ABN, BS, CLIA IgG, CLIA IgM, CLIA IgM-IgG, LFIA IgG, LFIA IgM, LFIA IgM-IgG, LFIA N protein, CMIA, and FIA) was performed through an sROC curve analysis (Figure 21) and due to implicit or explicit alterations between studies and variation in the cut-off points of the test, differences in sensitivity and specificity may occur [141,142]. The area under the curve (AUC) calculated for the diagnostic techniques for COVID-19 is shown in Figure 21, showing better performance for ABN. Furthermore, when the AUC was limited to the observed false positive rates (FPR) (AUC_FPR_), results revealed the relatively better performance of the ABN diagnostic test for COVID-19 (Figure 21).

## 4. Discussion

The disease COVID-19 has had a catastrophic effect, teaching future generations to examine several conditions that encourage the growth of infectious diseases [143]. The COVID-19 pandemic brought to light the gaps in disease detection, warning, and response systems. Given how interconnected the globe is and how quickly a pandemic might spread, it illustrated the necessity of restarting the global health and health security system [144]. The key factor in the spread of infectious diseases is human migration, and this factor is increasing due to globalization and transportation networks. In this environment, there is the potential for the quick and challenging management of the spread of harmful germs like SARS-CoV-2 [145]. Asymptomatic carriers may be able to transfer COVID-19 during the incubation period without displaying any symptoms or signs because the percentage of asymptomatic individuals who test positive for COVID-19 ranges from 8.44 percent to 39.00 percent [146]. In contrast, the production of antibodies starts days after the onset of the infection. As a result, the start time of the potential infection should be taken into consideration to make an accurate diagnosis, keeping in mind that molecular tests will become less sensitive over time while serological tests will become more sensitive after a few days of infection [147]. These factors make the quick and precise diagnosis of individuals with COVID-19 infections crucial. In addition, diagnostic tests required processing in laboratories with sophisticated materials, leading to a longer turnaround time and several days needed for results to be available. Some of these obstacles were eventually overcome by newly designed assays that had analytical precision that is more precisely specified than prior assays [148,149].

Since SARS-CoV-2 is an RNA virus, it can be detected using any of the various molecular tests for RNA detection [150,151]. To use DNA detection techniques, reverse transcriptase must convert the virus RNA into a DNA complement [152]. Although RT-PCR is currently the most used molecular technique for the early identification of COVID-19, several promising alternatives exist, including RT-LAMP and CRISPR [153]. Due to its high specificity, RT-PCR is regarded as the gold standard molecular diagnostic test for COVID-19 throughout the world; however, the slow detection of the virus was caused by the limited availability of kits and reagents, the use of pricy laboratory equipment, and the requirement for qualified personnel [154,155]. The RT-PCR test has also been documented to fail in suspected and confirmed patients with clinical repercussions. In this situation, further clinical and molecular testing ought to be considered when determining the COVID-19 diagnosis [26,154]. The identification of asymptomatic infections with high specificity and sensitivity is a key point for managing the pandemic, however, because it has been reported that asymptomatic infected people have a few copies below the detection limit of the nucleic acid within the upper respiratory tract, it has to lead to false negatives in asymptomatic infected individuals [156]. The investigation revealed no discernible difference between RT-PCR and CRISPR for these parameters, with RT-PCR having a median sensitivity and specificity of 94.5 and 94.4 percent, respectively. Both PCR and CRISPR are used. CRISPR, however, outperformed AUCFPR when compared, which can be partially attributed to the disparity in the number of studies considered and sample sizes. The use of CRISPR to diagnose COVID-19 offers several benefits, including quick detection (around 30 min), high sensitivity and accuracy, mobility, and lack of requirement for specific laboratory equipment [155]. Additionally, RT-LAMP demonstrated among the molecular assays the lowest sensitivity (median, 91.9%) and the best specificity (median, 98.8%). RT-LAMP is superior to RT-PCR in that it can be amplified at a constant temperature without the use of a thermocycler. One week before the beginning of symptoms, SARS-CoV-2 infection can be detected using molecular diagnostics. Molecular diagnoses are crucial for the early detection of COVID-19 because antibodies can only be identified 8 days after the onset of symptoms [157,158]. In general, molecular tests perform better when compared to serological testing when used to diagnose COVID-19, with AUCFPR values of 0.949, 0.936, and 0.952 for RT-PCR, RT-LAMP, and CRISPR, respectively.

To find the antibody response brought on by COVID-19, there are numerous serological tests available. ELISA, CLIA, LFIA, CMIA, FIA, and ANB are the principal techniques [159,160,161]. The performance of serological assays for the identification of SARS-CoV2 has been reported to be satisfactory, however, this performance is dependent on the patient’s recovery, making it important to understand the kinetics of antibodies during SARS-CoV-2 infection. Establishing the serological result is crucial in this situation for illness diagnosis [161,162]. Serological assays based on ELISA, CLIA, LFIA, CMIA, and FIA data have modest sensitivity (median less than or equal to 90%) and high specificity (median higher than or equal to 97%). Since they cannot distinguish between individual immune responses or intrinsic immunological abnormalities, low sensitivity might result in false negative results. Additionally, it must be considered that antibodies can be generated in asymptomatic patients. Nevertheless, their titers are lower than those found in symptomatic patients [163]. The immune system is stimulated by SARS-CoV-2 invasion and antigen release to develop a variety of antibodies (IgM/IgA/IgG). Immune cells of the host produce IgM and IgA at an early stage of infection, whereas IgG is produced at a later stage [164,165,166].

Diagnostic methods such as ELISA, CLIA, and LFIA were classified based on the antibodies they identified (IgG, IgM, IgA, and IgG-IgM). These tests’ ability to detect IgG produced the highest AUC_FPR_, indicating the antibody’s superior performance. This may be because IgM levels begin to decline at week 5 and almost completely disappear by week 7, whilst IgG levels continue to rise after week 7, indicating the greater stability of IgG [167]. On the SARS-CoV-2 membrane, however, several structural proteins are anchored, primarily the spike (S), nucleocapsid (N), membrane (M), and envelope (E) [166,168]; N protein can be helpful in the diagnosis of COVID-19 since it is a highly immunogenic protein that is extensively produced during infection [169]. The median sensitivity and specificity of the LFIA approach were 74.7 percent and 99.4 percent, respectively, when its performance was compared to the detection of the N protein of SARS-CoV-2. Additionally, RNA is less stable than N protein, which can effectively make up for the limited sensitivity it exhibits [170]. The ability of numerous companies to produce serology tests, which may be administered to millions of people daily, can aid in the improvement of SARS-CoV-2 detection, particularly in nations with low resources [170]. However, it is advised to combine clinical, molecular, and serological diagnostic tests to achieve acceptable sensitivity and specificity [159].

It is important to note that the ANB diagnostic test had the highest sensitivity and specificity scores for the identification of COVID-19 when molecular and serological methods were considered. It also had the highest AUC_FPR_, coming in at 0.984. Neutralizing antibodies can shield cells from virus invasion and provide protective immunity; they are created weeks after infection [171]. ANBs can objectively detect SARS-CoV-2 neutralizing antibodies, allowing for the analysis of the correlation between neutralizing antibody levels and disease severity. They can also forecast the likelihood of reinfection in COVID-19 patients [171,172]. The plaque reduction neutralization test (PRNT), the current gold standard for serological assays and the assessment of immune protection, is typically used to evaluate neutralizing antibodies [173].

The genetic makeup of SARS-CoV-2 can be investigated using molecular techniques like NGS, enabling its discovery [174], and MA has been utilized for genotyping and the identification of agents that cause diseases like SARS-CoV-2 [175,176]. Additionally, COVID-19 can be detected using the ECLIA serological-based diagnostic methodology [177,178]. The number of chosen studies for these methodologies, however, hindered their inclusion in the meta-analysis because the meta-analysis requires at least five studies with a *p*-value of less than 0.05 [142]. For “COVID-19”, “Sensitivity and specificity”, “Next-generation sequencing”, “Microarray Analysis”, and “Immunoassay”, individual MeSH term searches revealed 195.931, 641.415, 49.966, 95.596, and 2.150 studies, respectively, but linking them turned up just 16, 3, and 367 research studies. For what ought to be regarded as work constraints, typical mistakes in systematic review and meta-analysis research, including study location and selection, missing crucial information about the results, improper subgroup analysis, conflicts with fresh experimental data, and duplicate publication [179]. Additionally, several issues, including the heterogeneity of the study groups, the clinical settings, and the diagnostic performance measures, were identified in the current investigation. Contrarily, inaccurate assessments of the results of diagnostic tests may result in an overestimation [142,180]. It should be remembered that combining clinical, molecular, and serological diagnostic tests is advised to achieve acceptable sensitivity and specificity [159,181].

## 5. Conclusions

For a pandemic to be effectively managed and its spread to be stopped, it is critical to accurately detect emerging infectious agents like SARS-CoV-2. In the current study, the effectiveness of various diagnostic techniques reported for COVID-19 was assessed. For the detection of SARS-CoV-2, molecular tests (RT-PCR, RT-LAMP, and CRISPR) performed better than serological tests (ELISA, CLIA, LFIA, CMIA, and FIA) in terms of sensitivity and specificity. Additionally, it was discovered that serological tests had a very poor sensitivity but a high specificity, particularly when IgG was found. It should be noted that the ANB-based diagnostic tool reported the best performance among all the investigated approaches, demonstrating the potential for the diagnosis of SARS-CoV-2 infection. IgM and IgG serological diagnostic assays could be introduced based on the findings to track the COVID-19 acute phase and conduct ongoing surveillance. These tests can aid in identifying the existence of certain antibodies against the SARS-CoV-2 virus in the human body, which is helpful for detecting the illness when it is still in its acute stage. They may also be helpful in identifying those who have already contracted the virus and are immune to it. However, there is still potential for improvement in testing generally, and emphasis should be given to the creation of quick, scalable, and accurate assays for the prevention of future SARS-CoV-2 epidemics and other infectious diseases that might emerge.

## Data Availability

Not applicable.

## Supplementary Materials

The following supporting information can be downloaded at: https://www.mdpi.com/article/10.3390/diagnostics13091549/s1, Figure S1: Study data and paired forest plot of the Positive Likelihood ratio, Negative likelihood ratio, and Diagnostic Odds ratio of reverse transcription–polymerase chain reaction (RT-PCR) in the diagnosis of COVID-19; Figure S2: Study data and paired forest plot of the Positive Likelihood ratio, Negative likelihood ratio, and Diagnostic Odds ratio of reverse transcriptase loop-mediated isothermal amplification (RT-LAMP) in the diagnosis of COVID-19; Figure S3: Study data and paired forest plot of the Positive Likelihood ratio, Negative likelihood ratio, and Diagnostic Odds ratio of clustered regularly interspaced short palindromic repeats (CRISPR) in the diagnosis of COVID-19; Figure S4: Study data and paired forest plot of the Positive Likelihood ratio, Negative likelihood ratio, and Diagnostic Odds ratio of enzyme-linked immunosorbent assay (ELISA) for IgG in the diagnosis of COVID-19; Figure S5: Study data and paired forest plot of the Positive Likelihood ratio, Negative likelihood ratio, and Diagnostic Odds ratio of enzyme-linked immunosorbent assay (ELISA) for IgM in the diagnosis of COVID-19; Figure S6: Study data and paired forest plot of the Positive Likelihood ratio, Negative likelihood ratio, and Diagnostic Odds ratio of enzyme-linked immunosorbent assay (ELISA) for IgA in the diagnosis of COVID-19; Figure S7: Study data and paired forest plot of the Positive Likelihood ratio, Negative likelihood ratio, and Diagnostic Odds ratio of antiviral neutralization bioassay (ANB) in the diagnosis of COVID-19; Figure S8: Study data and paired forest plot of the Positive Likelihood ratio, Negative likelihood ratio, and Diagnostic Odds ratio of biosensors (BS) in the diagnosis of COVID-19; Figure S9: Study data and paired forest plot of the Positive Likelihood ratio, Negative likelihood ratio, and Diagnostic Odds ratio of chemiluminescence immunoassay (CLIA) for IgG in the diagnosis of COVID-19; Figure S10: Study data and paired forest plot of the Positive Likelihood ratio, Negative likelihood ratio, and Diagnostic Odds ratio of chemiluminescence immunoassay (CLIA) for IgM in the diagnosis of COVID-19; Figure S11: Study data and paired forest plot of the Positive Likelihood ratio, Negative likelihood ratio, and Diagnostic Odds ratio of chemiluminescence immunoassay (CLIA) for IgM-IgG in the diagnosis of COVID-19; Figure S12: Study data and paired forest plot of the Positive Likelihood ratio, Negative likelihood ratio, and Diagnostic Odds ratio of lateral flow immunoassay (LFIA) for IgG in the diagnosis of COVID-19; Figure S13: Study data and paired forest plot of the Positive Likelihood ratio, Negative likelihood ratio, and Diagnostic Odds ratio of lateral flow immunoassay (LFIA) for IgM in the diagnosis of COVID-19; Figure S14: Study data and paired forest plot of the Positive Likelihood ratio, Negative likelihood ratio, and Diagnostic Odds ratio of lateral flow immunoassay (LFIA) for IgM-IgG in the diagnosis of COVID-19; Figure S15: Study data and paired forest plot of the Positive Likelihood ratio, Negative likelihood ratio, and Diagnostic Odds ratio of lateral flow immunoassay (LFIA) for N-protein in the diagnosis of COVID-19; Figure S16: Study data and paired forest plot of the Positive Likelihood ratio, Negative likelihood ratio, and Diagnostic Odds ratio of chemiluminescent microparticle immunoassay (CMIA) in the diagnosis of COVID-19; Figure S17: Study data and paired forest plot of the Positive Likelihood ratio, Negative likelihood ratio, and Diagnostic Odds ratio of fluorescence immunoassay (FIA) in the diagnosis of COVID-19; Table S1: PRISMA 2020 Checklist.

## Abbreviations

The following abbreviations are used in this study.

ACE2	Angiotensin-converting enzyme 2
ANB	Antiviral neutralization bioassay
AUC	Area Under the Curve
AUC_FPR_	Area Under the Curve Restricted to The False Positive Rates
BS	Biosensors
CI	Confidence interval
CLIA	Chemiluminescence immunoassay
CMIA	Chemiluminescent microparticle immunoassay
COVID-19	Coronavirus disease
CRISPR	Clustered regularly interspaced short palindromic repeats
DOR	Diagnostic Likelihood Ratio
ECLIA	Electrochemiluminescence immunoassay
ELISA	Enzyme-Linked Immunosorbent Assay
FIA	Fluorescence immunoassay
FN	False negatives
FP	False positives
IgA	Immunoglobulin A
IgG	Immunoglobulin G
IgM	Immunoglobulin M
INPLASY	International Platform of Registered Systematic Review and Meta-analysis Protocols
LFIA	Lateral flow immunoassay
LR−	Negative Likelihood Ratio
LR+	Positive Likelihood Ratio
MA	Microarrays
MeSH	Medical Subject Headings
NCBI	National Center for Biotechnology Information
NGS	Next-generation sequencing
PRISMA	Preferred Reporting Items for Systematic Reviews and Meta-Analyses
PRNT	Plaque Reduction Neutralization Test
ROC	Receiver Operating Characteristics
RT-LAMP	Reverse transcriptase loop-mediated isothermal amplification
RT-PCR	Reverse transcription–polymerase chain reaction
SARS-CoV-2	Severe acute respiratory syndrome coronavirus 2
Se	Sensibility
Sp	Specificity
sROC	Summary Receiver Operating Characteristics
TN	True negatives
TP	True positives
WHO	World Health Organization
